# *Letter:* A Strategic Path Forward for Hospice and Palliative Care Must Focus on Equity: A Response to Byock

**DOI:** 10.1177/10966218251370702

**Published:** 2025-08-21

**Authors:** Karen Bullock, Ramona L. Rhodes, Marisette Hansan, Kimberly S. Johnson

**Affiliations:** ^1^ School of Social Work, Global Public Health. Boston College, Chestnut Hill, Massachusetts, USA.; ^2^ Department of Internal Medicine, UT Southwestern Medical Center, Dallas, Texas, USA.; ^3^ Peter O’Donnell Jr. School of Public Health, UT Southwestern Medical Center, Dallas, Texas, USA.; ^4^ LifeSprings Consulting Group, Columbia, South Carolina, USA.; ^5^ Division of Geriatrics, Department of Medicine, Duke University School of Medicine, Durham, North Carolina, USA.

*Dear Editor:*

In *“A Strategic Path Forward for Hospice and Palliative Care: A White Paper on the Potential Future of the Field”*,^1^ Dr. Byock offers an important call to action. His four-part framework—establishing clear standards, improving access to data, encouraging quality-based competition, and embracing the authentic brand of expert care—lays out a roadmap to improve serious illness care. He argues, “this is the moment for this vitally needed clinical specialty and its associated business community to embrace uncomfortable truths and chart a strategic path forward.” We agree. However, one of the most urgent uncomfortable truths is briefly and incompletely acknowledged in the article (2 sentences on p. 311). That is, not only do historically marginalized communities face unequal access to the benefits and progress of our field; they endure a disproportionate share of practices that lead to poor quality care,^2,3^ including many of the challenges that Byock highlights. They are the least likely to use hospice, and when they use it, are more likely to receive hospice from for-profit entities, face significant risk of predatory hospice business practices, receive lower quality hospice care, and may have less access to specialty palliative care.^4^

In today’s climate, where diversity, equity, and inclusion are being actively dismantled and the histories and voices of marginalized communities are under threat—a strategic path forward must be intentionally grounded in equity. We offer perspectives aligned with Byock’s framework through this lens.

*First, standards must embed cultural and structural competence*. Quality cannot be defined without a focus on equity. Standards should set expectations for culturally responsive care and spiritually respectful care, attention to social drivers of health, language access, and active community engagement.^5,6^ In addition, as we aim to advance the field more broadly, we must implement interventions that engage historically underrepresented populations.

*Second, data must expose disparities and drive accountability*. We support the call for transparent, accessible data, but it must be disaggregated by race, ethnicity, geography, and other social drivers of health. Without this granularity, inequities remain obscured. Data must also inform corrective action and equity-focused outcomes.

*Third, quality-based competition must not deepen disparities*. While competition may improve care for some, it may worsen outcomes for marginalized groups. Studies show that for-profit hospice providers serving these communities tend to deliver fewer services, especially near the end of life. Metrics must reward equity-driven innovation and inclusion. At the same time, we must realize that “choice” of providers is a luxury that many marginalized communities may not have. As such, regulatory agencies must monitor care quality and develop consequences for substandard care.

*Fourth, rebranding the field must include building trust*. We appreciate Byock’s call for the field to rebrand as specialists managing symptoms and fostering well-being. This is especially important since the field’s origins were shaped by cultural and clinical movements that largely excluded the voices and needs of marginalized communities. Any rebranding efforts must consider this reality and should reflect meaningful change in how care is not only discussed but also delivered.

We applaud Byock’s call to action.^1^ However, without an intentional focus on equity, for those who already experience care that is lower quality, less accessible, and less congruent with their values, any “new” plan will likely yield more of the same.^2,7,8^ In this context, we reflect on the article’s title. Although *“White Paper”* is traditionally associated with recommendations for policy change, the phrase may unintentionally suggest whose perspectives are centered—and whose are left out.^7^ Thus, a strategic path forward that improves care for all patients with serious illness and their families must employ an equity lens in everything we say, write, and do.
